# Notes and Comments

**Published:** 1923-06

**Authors:** 


### NOTES AND COMMENTS.

### VOLUNTARY HOSPITALS AND PAID DOCTORS.

AT the representative meeting of the British Medical Association, which is to be held at Portsmouth in July, the vexed question of payments to what have hitherto been the honorary medical staffs of voluntary hospitals will come up for further discussion and, apparently, for final decision. By that time the subject will have been under debate for two years and a half, and both doctors and hospital managements will be glad to have it settled. The Cave Committee, it will be remembered, advised that where the full cost of maintenance and treatment is paid by the patient, or by some authority on his behalf, a percentage of these payments should be placed to the credit of a staff fund, to be disposed of as each medical staff may decide. There has been, and still is, much difference of opinion, but, on the whole, the doctors have favoured some such course, and the Voluntary Hospitals Commission has defined a " Voluntary Hospital " not as necessarily an institution where medical treatment is free, but as one " managed by a voluntary committee and wholly or mainly supported from voluntary sources." In forming an opinion upon this difficult question it is essential to bear in mind that hospitals are rapidly changing their character in that the principle of payment in whole or in part by, or on behalf of, patients is being widely accepted. Their voluntary character is a precious possession which must on no account be endangered, but where the payments are on such a scale that the patient costs the hospital nothing, it is surely unreasonable to expect the medical staff to give its services free. It will never grow rich from this source, nor would it wish to do so. The experience and reputation which are gained by hospital practice are of far greater importance than money; but it would be a disservice to the public to encourage the notion that all hospital treatment ought to be eleemosynary.

### THE FUTURE OF HEALTH INSURANCE.

FOR the moment we do not propose to inquire whether the attacks upon the panel system which are so frequent and so violent just now are well or ill founded. We merely register the fact that, rightly or wrongly, dissatisfaction with the system is widespread, and elsewhere in our issue this month we place in piquant juxtaposition the condemnation of a London coroner and the reply of the British Medical Association. In this, as in all other controversies, much depends upon the point of view, and it is probable that the truth lies between the extraordinarily divergent views represented by those who can see little good, and those who can perceive little ill, in the panel system. That there are perfunctory, and even inefficient, panel doctors is patent; it is equally obvious that the bulk of them are neither the one nor the other. But there is danger in expecting too much, and we have ourselves very recently made suggestions with regard to laboratory service and the increase of facilities for consultation which would do much to remove such dissatisfaction as is justifiable, and in his second article on "Panel Pay and Extended Services" this month, Mr. R. W. Harris has some cogent things to say in this connexion. That there must, before long, be some reconsideration of certain details of the system is inevitable. In some respects it is a wasteful system. Tens of thousands of persons who are compulsorily insured neither derive, nor intend to derive, any benefit from their payments. They are irritated by their inclusion and, since they are not inscribed upon any panel, their money and their employers' money is, in a sense, wasted. It is arguable that they are suffering from an insufficiency of chastened altruism; but the whole subject deserves, and requires, fair and reasonable discussion. Unmeasured attacks are as unwise as thorough-going defence. This is a question for the community as well as for the doctors. The one has to be assured that it is obtaining first-rate medical treatment—anything less than that is not worth having; the doctors have to feel assured that they are being fairly treated, and that they are not being made scapegoats for the defects of a system. Meanwhile, let us not forget that in the decade which has elapsed since Health Insurance was launched there has been a notable and progressive improvement in the public health. The one may not be the consequence of the other; but it would be unjust to ignore a notorious fact.

### OCCUPATIONAL THERAPY.

ATTENTION in America is again being called to the great influence in curing, or at least relieving, pathological conditions which can be exerted by occupations particularly suited to the sufferers. It is, of course, easier in that country to apply such methods upon organised lines ecause of the great institutional scope available and, in recent times, the relative absence of financial depression. The term itself i[illegible] almost self-explanatory, but, in order to remind us that we ourselves are not altogether forgetful of the possibilities, it may be remarked that many o the controlled occupations followed by our sanatorium patients—and even the activities displayed in many excellent hospital, massage and remedial exercise departments—approach closely to the beginnings of true occupational therapy. There can be little doubt that, whatever the patient's ailment, provided it is not severe enough to require rest or treatment in bed, there must be some occupational circumstance in which his chance of recovery is greatest. The new science involves c[illegible]eful and organised investigations to find the exact conditions most benefi ial to each mind—satisfying to them and available without utterly destroying their utility as active members of the community. It must not be assumed that such treatment can work other than as a direct appendix to exact medical diagnosis. Never can correct occupational therapy produce cure-alls. The whole aim is, the exact nature of the disease, physical or mental, being determined, to suit the circumstances to it and to the study of these circumstances much of the new work must be devoted. It is impossible in a short note to touch on the infinite variety of occupations available, but if we remember how many of our patients recuperate amazingly if they can only be led to take up special interests, in short, to occupy their minds; or how many minor ailments became progressively less frequent after the taking up of golf then, we have a first glimpse of the elements of occupational therapy. With it a new field should be opened for many in the general cause of health.

### THE MIDDLE CLASSES IN HOSPITAL.

OUR leading article of last month on this subject has produced an interesting letter from Mr. C. E. A. Bedwell, the House Governor of King's College Hospital, which has in successful operation an arrangement for the reception of paying patients. It is not surprising to learn that the wards for private patients are greatly appreciated, and that many a person who could not by any possibility be described as "poor" in the hospital sense of the word, has been saved by the existence of those wards from what Mr. Bedwell rightly calls the "grave financial calamity" which serious illness would otherwise have entailed upon them. Subscribers in financial need who make use of these special wards can claim to have their subscriptions for a certain time past deducted from the charge for the maintenance of private patients. There is abundant room for a wide extension of schemes for making the hospitals available for those who have hitherto had little opportunity of using them, and we await with keen interest the working out of Sir Napier Burnett's promised scheme of insurance which shall enable the middle and upper classes to enjoy the "inestimable benefits" of hospital treatment.

### MEDICAL TEACHING REFORMED.

AT the beginning of this year so complete a revision of the medical curriculum came into force that the change affects the student even before he is actually registered as particularly devoting his energies to medical science. No stage of the educative process in his medical school or right through his hospital career remains unaffected, and no change could be fraught with greater meaning to the profession of the future. Generally speaking the call for these developments was well understood by the doctors themselves, and, to them, they represent simply a natural well considered process of improvement. But their critics have of late been a little too prone to see in the reformation an admission of weakness and of wasted opportunity. Therefore the appearance of a memorandum by Sir George Newman on "Recent Advances in Medical Education in England" (Stationery Office, 1s. 3d.) is doubly welcome, because the very able writer provides not only an explanation which is also a complete justification of the changes, but a work which may well be inspected by all interested in education, particularly those unkindly critical of past medical ways. It is commonly expected that the doctor should possess, in marked degree, power to appreciate the difficulties of others. Perhaps it is not too much to ask others to appreciate his. In any case, study of this work will make many things clear, and provide means for better public perception of the present training of the doctor. As Sir George says, when one reads a statement of this kind one is apt to gain the impression that the medical curriculum may impose a burden too heavy to be borne, but the revisions imply compensations, too. The number of systematic lectures which the student must attend has been substantially reduced. Though they are just as discriminating, the form of the examinations to which he must be subjected is far less burdensome. He has, after all, five years in which to accomplish everything that we can expect. This everything, as Sir George Newman wisely holds, is only "to provide himself with tools of learning in order that by experience he may become a reliable and effective workman." Train the student rightly and later, as a doctor, he will develop his own resources. This memorandum makes it clearer than ever that there is much in the study of medicine besides mere "qualification."

### THE INTRUDING CURRANT.

THE daily newspapers have recorded that at his recent auction sale of Large Black pigs at Lydney Park, Lord Bledisloe provided a lunch which, with the exception of the currants in the cake, was entirely produced on his own farms, from the pork, mutton, and venison to the cider. It was a sad pity to spoil the symmetry of this "example in home production" by permitting the intrusion of currants. The taste for currants is somewhat of a mystery. They are not especially alluring to eat—one sultana is worth a hundredweight of them—and no inconsiderable proportion of people find them indigestible. Cider is nectar and venison ambrosia, and to mix these gifts of the gods with mere currants is to show a lack of appreciation of the fitness of things which is shocking in more senses than one. We are quite sure that Lord Bledisloe does not feed his Large Black pigs (at £25 each) upon currants.

### SIR SHIRLEY MURPHY.

EVEN had he not been the first Medical Officer of the London County Council—he held that great post for nearly a quarter of a century—the late Sir Shirley Murphy would still have made an outstanding career in public health. But his appointment as the first Medical Officer of the new authority gave him an unexampled opportunity of putting into execution the large and sound ideals on the subject of public health which had informed his mind from an early stage of his medical career. While yet a very young man he became a recognised authority upon the subjects with which, at a later date, he was called upon to deal on a great scale. The clearance of slum areas, the influence of dense populations on infectious diseases, problems of water supply, the contamination of foods—upon these, and many other difficult and complicated questions of sanitary administration, Sir Shirley Murphy was not only an expert, but in some respects a pioneer. During his time at Spring Gardens the death-rate of Inner London declined substantially, and in every respect he left the health of the Metropolis vastly ameliorated. Like so many other distinguished medical men and sanatarians he gave of his best to his country during the war, when there fell upon him the heavy responsibility of the charge of the sanitary services of the London area. And it is pleasant to remember that Sir S. H. Hamer, the present incumbent of his County Council officer, is the son of one of Si Shirley Murphy's oldest friends.

### THE BURDEN OF THE NEUROTIC.

"NEUROSIS," says the writer of the article, "Wasting Money on the Neurotic," which we print this month, is "one of the great national problems." Looking at the enormous cost of dealing with this widespread and elusive trouble, no one is likely to quarrel with that statement. The difficulty of dealing with it is obvious, since, as the writer points out, we have first to be sure that we are really faced with a neurotic condition and not with organic disease. Difficulties, however, are made to be overcome, and although neurology may still be in its infancy and we may yet be in the stage of collecting information, we know far more about an affection which poisons innumerable lives than we knew a few years ago. Meanwhile the medical man is easily deceived, and is especially in need, in this connexion, of specialist aid in diagnosis, and our contributor applauds the suggestion already made in these pages for the establishment of central consulting rooms. That would be a distinct step forward, but the whole subject is complicated by the fact that we are dealing not so much with disease as with human nature, which is the most intractable of all diseases. Doctors have to deal with that as it is, and not as doctors would have it. Our contributor's view of neurosis may to many seem rather severe, and those unfamiliar with modern psychology might with advantage read Mr. Bernard Hart's little book on "The Psychology of Insanity" and follow it up with Mr. Curdy's "War Neurosis." The civilian application of the same principles is best illustrated by the publications of some of the American psychological clinics.

### ALCOHOL AND LIVER DISEASE.

CIRRHOSIS of the liver—the "hobnail" liver—is so often due to chronic alcoholism that the sobriety of a community may, to a certain extent, be gauged by the number of "hobnails" with which it is studded. This point is well brought out in a recent statistical study by a Dane, Dr. Harald Jacobsen. In April, 1917, a certain degree of Prohibition was introduced into Denmark. In the previous year the consumption of alcohol, reckoned as 100 per cent. alcohol, was 6·45 litres per head, beer, wine and spirits being included in this calculation. This figure fell to 3·15 in 1917, and to 1·5 in 1918. In 1920 it showed a considerable rise, being 2·8 litres of 100 per cent. alcohol per head. During 1916 the Communal Hospitals in Copenhagen treated 124 cases of cirrhosis of the liver. In 1917 this figure fell to 92, in 1918 to 49 and in 1919 to 28. In 1920 it rose to 45. It will thus be seen that the parallelism of alcohol consumption and cirrhosis of the liver was remarkably uniform. The evidence of the post mortem room told the same tale as the hospital wards. In 1916 cirrhosis of the liver was the cause of death in 2·9 per cent. of all the persons coming to necropsy. In 1919 this ratio was reduced to 0·7. Dr. Jacobsen's figures thus show that the incidence of this disease of the liver falls abruptly with the introduction of Prohibition, and he is therefore probably correct in concluding that it may be aborted even in those persons who, till the introduction of Prohibition, had for many years been steadily qualifying themselves for inclusion among the "hobnails." It would be interesting to learn if pathologists in the United States have come to the same conclusion as Dr. Jacobsen with regard to the effects of abstemiousness on the incidence of the disease.

### MIRACULOUS CUTANEOUS INJECTION.

DR. JENNER and Baron Münchhausen were both great men. Both contributed to the happiness of the human race, but in different ways and in different degrees. Though they represented very different types of genius, both certainly showed genius, and they had one quality in common—that of imagination. After all, the gulf is not so very wide between a Wilbur Wright and an H. G. Wells. And who shall say that the prophetic vision of the latter contributed less to the science of aviation than the mechanical genius of the former? A very remarkable book has just been published in Germany by Dr. W. Ponndorf whose therapeutic claims verge on the miraculous. If posterity endorses them, posterity will also class Dr. Ponndorf with Jenner and Pasteur. Dr. Ponndorf believes that, by scratching the skin and rubbing pure tuberculin or a mixture of tuberculin with some other vaccine into the abrasions, it is possible to cure practically every disease to which man is heir. Apart from fractures, cancer, and a few other diseases, he regards almost every morbid process as the product of the tubercle bacillus and its allies—the germs of mixed infection. So, whether a patient is suffering from pure tuberculosis or scrofula, asthma, Graves's disease, rheumatism, epilepsy or melancholia, he rubs in one or other of his vaccines. His book is full of records of almost uninterrupted successes, achieved in an almost infinite variety of diseases. It is this variety that puzzles and breeds scepticism. The idea that the same key should fit so many locks is a little startling. Dr. Ponndorf states that he has treated about 5,000 patients with cutaneous inoculation, and as he has a large following in Germany, there should soon be an appreciable reduction in the German death rate if his claims are justified. All things considered, it would probably be best to await confirmation of Dr. Ponndorf's claims before applying his treatment wholesale to patients in this country.

### BACK-TO-BACK.

OUR Special Commissioner who this month describes the result of his inquiries into the health conditions of the city of Bradford, tells us that this great industrial centre contains 40,000 houses of the body and soul destroying back-to-back type. He points out how certain it is that these hopeless dwellings will continue to fill the hospitals and churchyards and to depress the general level of health in the city. Happily those who are responsible for the sanitary welfare of Bradford are alive to the dangers, and are doing what is immediately possible to reduce them. But the problem is so enormous that it is impossible to make any serious impression upon it within a reasonable time. The slums cannot be deliberately burned down, nor does it follow that every back-to-back house is in a slum. The coming legislation affecting crowded and unhealthy areas may help, but it is clearly necessary that in Bradford and other towns similarly situated great homogeneous clearance schemes should be elaborated and carried out in sections as means become available. This is emphatically a case in which every little helps, but it is a peculiarly unhappy circumstance that the cost of clearance and re-housing should at present be so heavy that slow progress alone is possible. Meanwhile the growth of civic consciousness in Bradford may, we hope, be depended upon to insist that every reasonably practicable measure shall be put into operation to reduce the back-to-back enormity.

### POISON IN FURS.

AFTER a series of careful investigations by Dr. Semon and Mr. Skinner, of the Royal Northern Hospital, and others, the factor which makes some furs so remarkably irritating to the skin seems to be definitely determined. It is not the fur of any particular animal which is the cause of all the trouble, but simply the dye used in giving it tone. Of these tones as such it is hardly possible to select one that is more dangerous than another, the risk arising from insufficient fixing of the dye; therefore cheap furs are always the culprits. The dyes mainly concerned belong apparently to one of the metaphenylene diamine groups, which can be completely fixed in the fur by a bichromate mordant if the manufacturing process is carried out properly. If the fixation is not completely and carefully done, the dye being soluble in the fatty secretions and sweat of the skin, some of it is extracted during use and after repeated applications, and though the amount be small, a severe and lasting eczematous irritation of the skin results. The relation between cause and effect seems now finally established. Obviously it implies not only a general warning against the cheaper furs, but also supervision of the manufacturers' methods. In this latter respect, considering the high prices, and profits, too, presumably, still ruling, stringent measures might justifiably be taken.

### THE ROCKEFELLER FOUNDATION.

ON May 14 the Rockefeller Foundation kept its tenth birthday, and celebrated the occasion by giving an account of its stewardship of the millions entrusted to it by the benevolence and public spirit of Mr. J. D. Rockefeller. It has expended more than fifteen millions sterling—the whole of its income—and three millions and a half of its capital as well. As it has felt its feet it has concentrated upon public health and medical education, and upon the first it has spent more than three and a half millions and upon the second nearly five millions. It is a noble record, and the work has been greatly helped by the International Health Board, a sub-infeudation of the Foundation, which seeks to promote public health throughout the world by demonstrating the methods and cost of controlling certain diseases, notably malaria, yellow fever, and hookworm disease, by fostering the growth of Governmental health agencies, and by the formation of schools of hygiene. London has had its share of the vast sums that have been devoted to these agencies for the promotion of the public health—agencies which have accomplished an enormous amount of splendid work for the good of the human race, and will, we may trust, yet accomplish still greater things.

### THE CHIEF CAUSES OF DEATH IN NEW YORK.

DO the remarkable changes which, according to vital statistics, sometimes occur in the space of only a dozen years indicate *bona fide* changes in the behaviour of certain diseases, or have new diagnostic methods and revised conceptions of pathology made us attach new values to comparatively unalterable conditions? In 1910 organic heart disease in New York occupied the third position as the cause of death, the actual figures being 6,780. In 1922 the deaths from organic heart disease had risen to 13,370, constituting the most frequent cause of death. In 1910 the death-rate from cardiac diseases was only 144 per 100,000, and in 1922 it was as high as 229. Is it possible that the actual incidence of fatal heart disease can have increased so enormously in only a score of years? It is easier to accept the statistics for fatal tuberculosis because the decline in the tuberculosis death-rate is a well-established phenomenon in many other parts of the world over the same period. In 1910 tuberculosis occupied the second place as a leading cause of death in New York; in 1922 it was relegated to the fourth place. In 1910 tuberculosis caused 10,074 deaths, and in 1922 only 5,794 deaths, although the population had increased in the interval by a million. The actual death-rate from tuberculosis per 100,000 were reduced from 210 to 99. It will thus be seen that the improvement in the tuberculosis death-rate has almost wiped out the debit booked under the heading of organic heart disease. Were we to take these figures at their face value, the mortality from the most important diseases would seem to fluctuate as wildly as the most notoriously speculative securities on the Stock Exchange, but in mortality returns, as well as in other spheres, the statistician may ultimately prove to have been artlessly romancing.

